# Supplementary figures and images for: Genome editing in the unicellular holozoan Capsaspora owczarzaki suggests a premetazoan role for the Hippo pathway in multicellular morphogenesis
Source: eLife. 2022 Jun 6;11:e77598. doi: 10.7554/eLife.77598 (PMC9170242; doi:10.7554/eLife.77598)

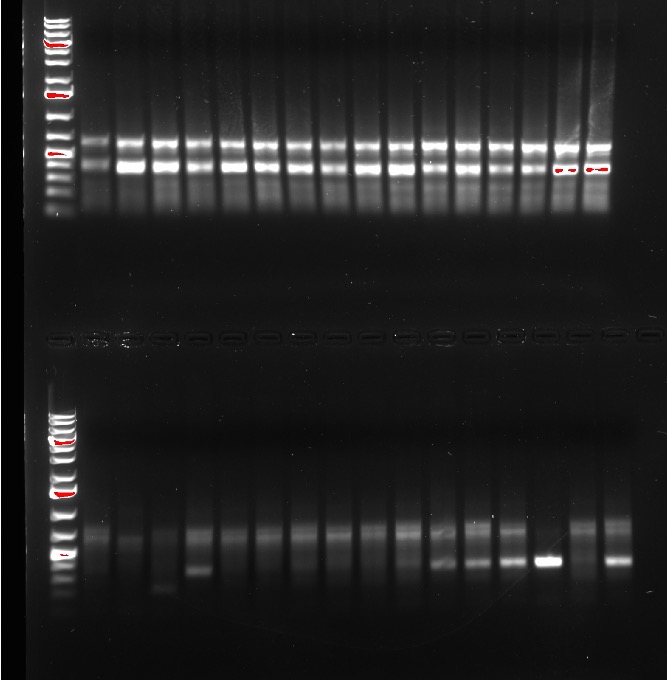

Supplement: Figure 1—figure supplement 2—source data 1. [file elife-77598-fig1-figsupp2-data1.zip › Figure 1-supplement 2-source data 1.tif]

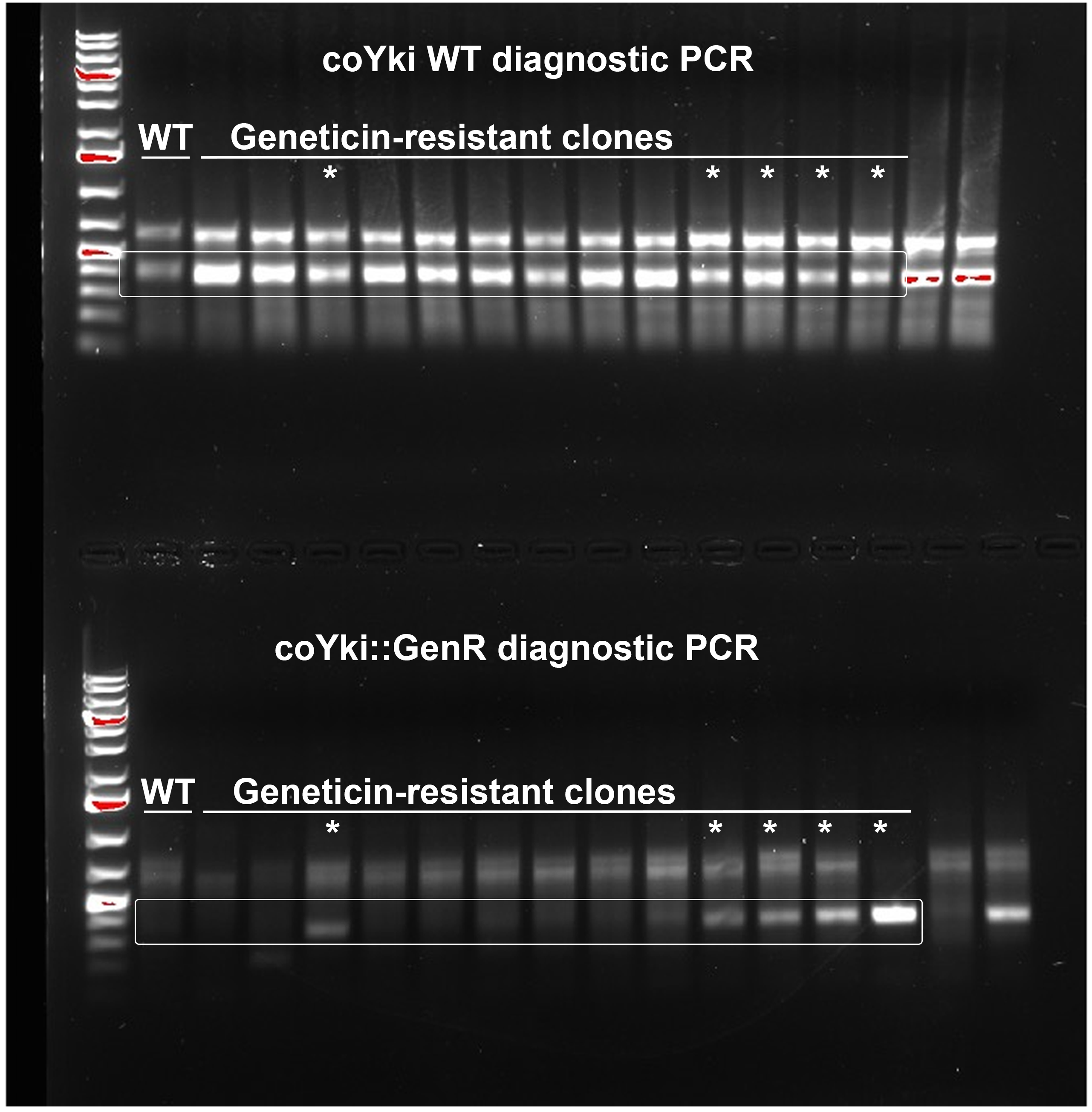

Supplement: Figure 1—figure supplement 2—source data 2. [file elife-77598-fig1-figsupp2-data2.zip › Figure 1-supplement 2-source data 2.tif]

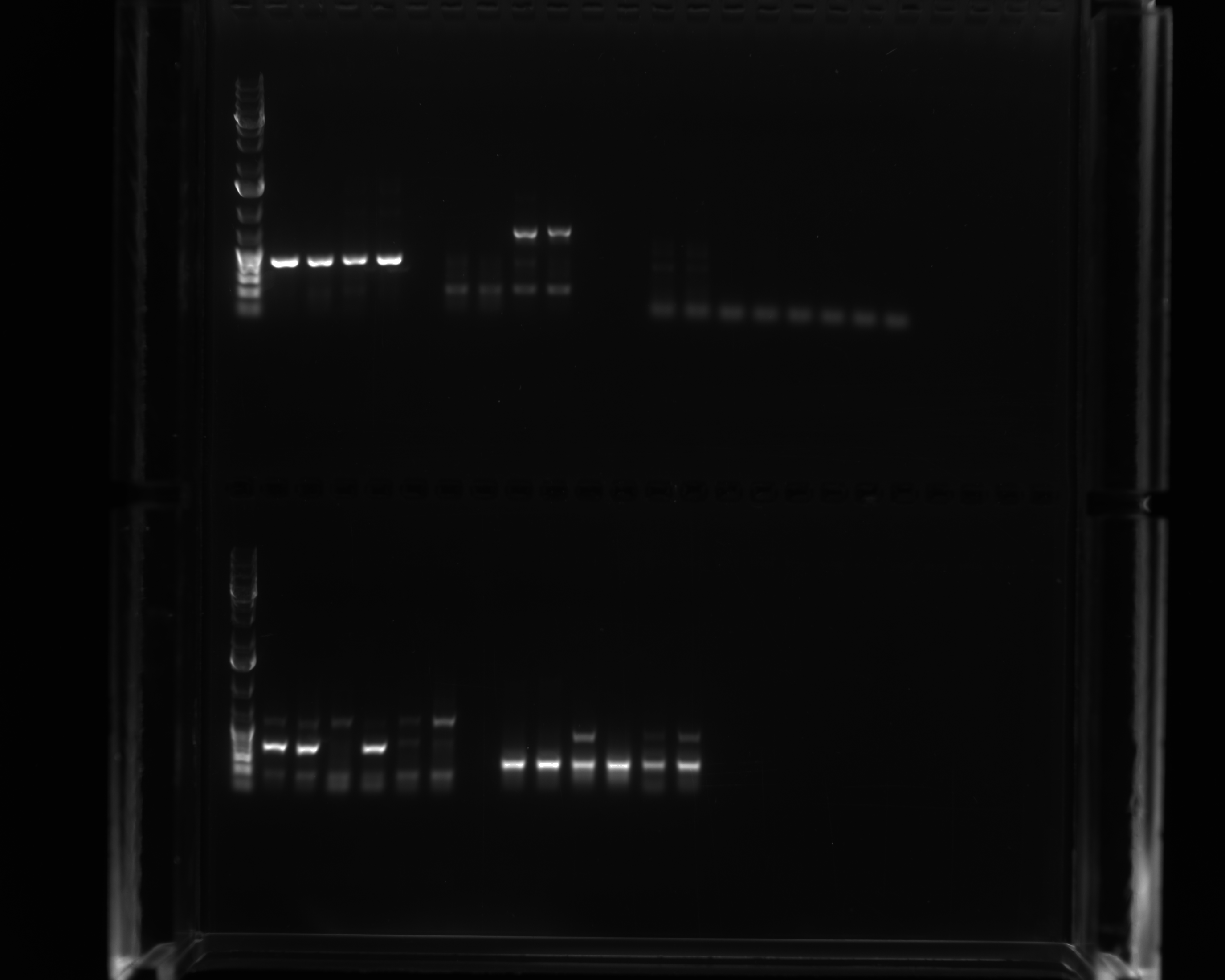

Supplement: Figure 1—figure supplement 2—source data 3. [file elife-77598-fig1-figsupp2-data3.zip › Figure 1-supplement 2-source data 3.tif]

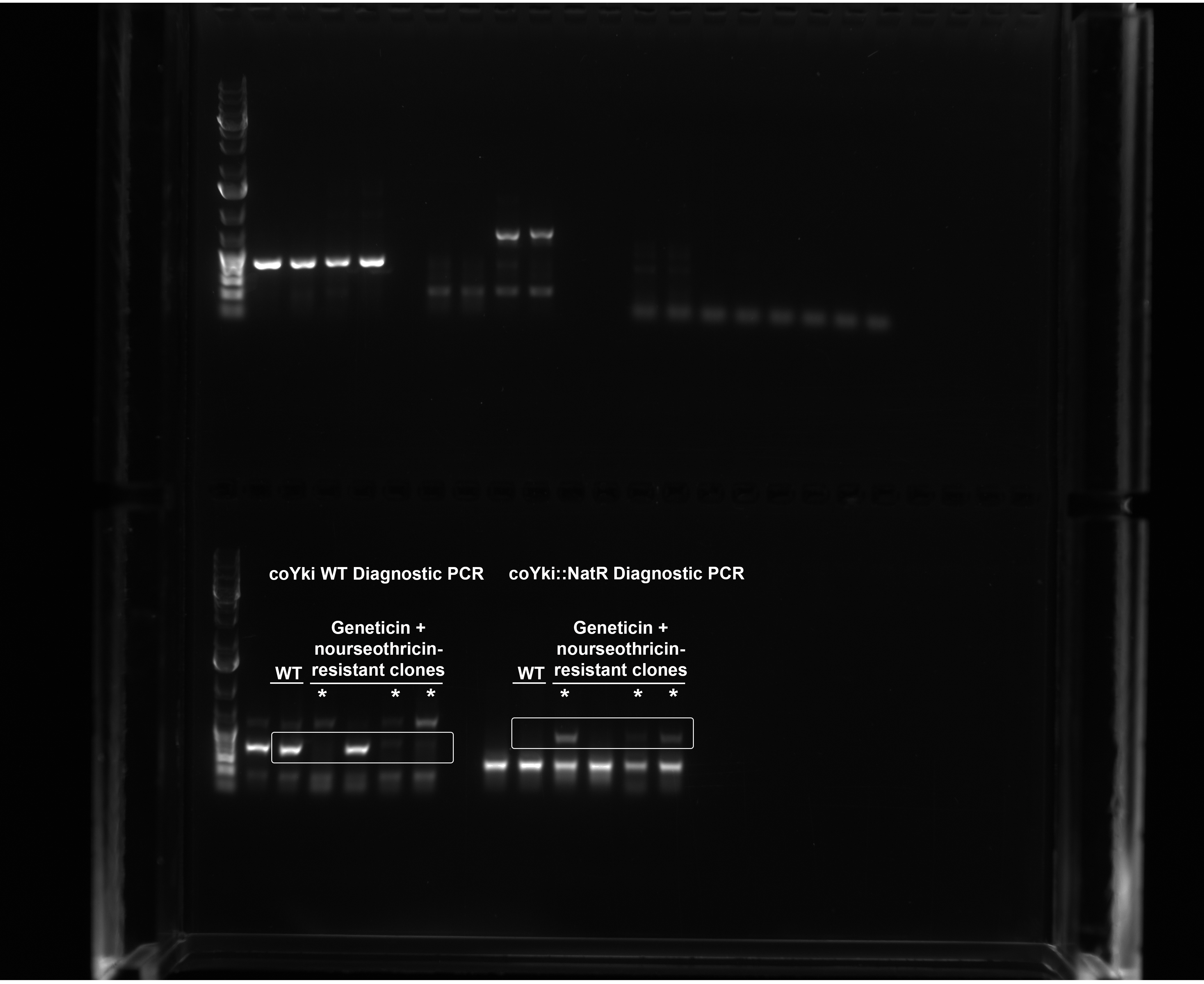

Supplement: Figure 1—figure supplement 2—source data 4. [file elife-77598-fig1-figsupp2-data4.zip › Figure 1-supplement 2-source data 4.tif]

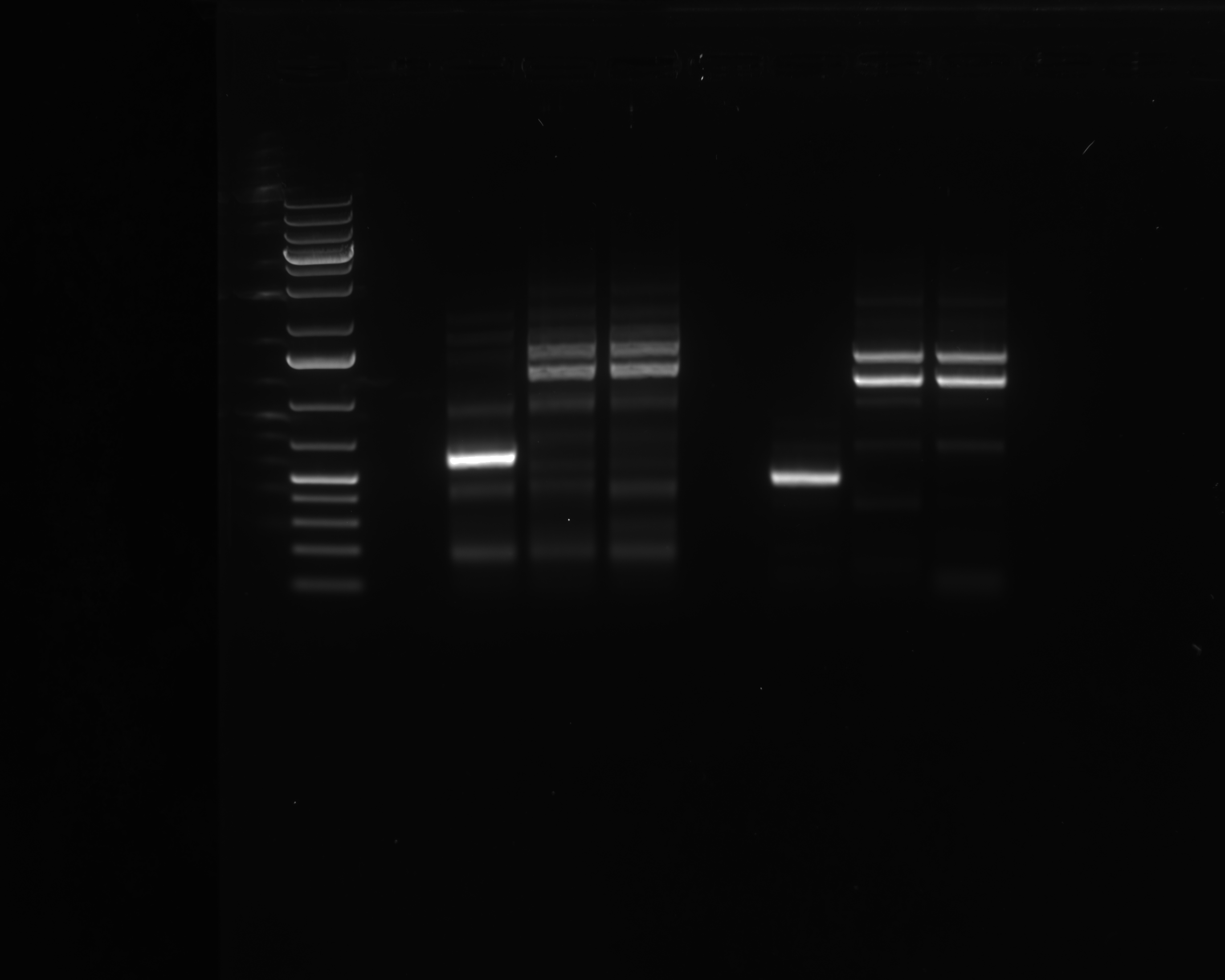

Supplement: Figure 1—figure supplement 3—source data 1. [file elife-77598-fig1-figsupp3-data1.zip › Figure 1-supplement 3-source data 1.tif]

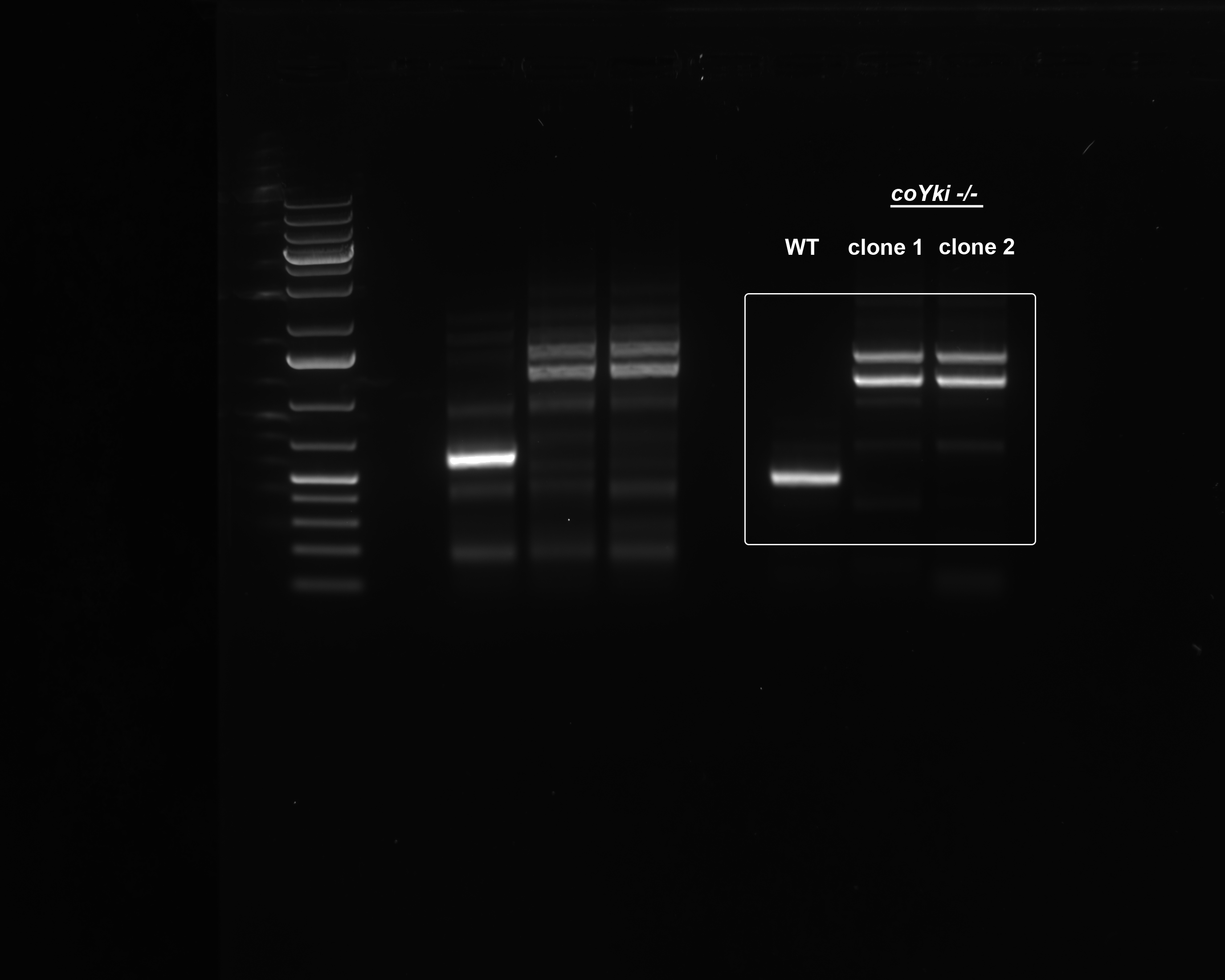

Supplement: Figure 1—figure supplement 3—source data 2. [file elife-77598-fig1-figsupp3-data2.zip › Figure 1-supplement 3-source data 2.tif]
